# Implementing community based inclusive development for people with disability in Latin America: a mixed methods perspective on prioritized needs and lessons learned

**DOI:** 10.1186/s12939-023-01966-8

**Published:** 2023-08-04

**Authors:** Andreas Bachfischer, Martha Cecilia Barbosa, Angel Alberto Riveras Rojas, Reinaldo Bechler, Eva-Maria Schwienhorst-Stich, Christa Kasang, Anne Simmenroth, Sandra Parisi

**Affiliations:** 1 Department of General Practice, Institut Für Allgemeinmedizin, University Hospital Würzburg, Haus D7, Josef-Schneider-Straße 2, 97080 Würzburg, Germany; 2 DAHW Latin America, GLRA German Leprosy and Tuberculosis Relief Association, Bogotá, Colombia; 3 DAHW HQ, GLRA German Leprosy and Tuberculosis Relief Association, Würzburg, Germany

**Keywords:** Community participation, Peer support, Leprosy, Community leader, Community based rehabilitation, South America, Empowerment, Participative implementation research, Work inclusion, Health access

## Abstract

**Background:**

Research on the needs of people with disability is scarce, which promotes inadequate programs. Community Based Inclusive Development interventions aim to promote rights but demand a high level of community participation. This study aimed to identify prioritized needs as well as lessons learned for successful project implementation in different Latin American communities.

**Methods:**

This study was based on a Community Based Inclusive Development project conducted from 2018 to 2021 led by a Columbian team in Columbia, Brazil and Bolivia. Within a sequential mixed methods design, we first retrospectively analyzed the project baseline data and then conducted Focus Group Discussions, together with ratings of community participation levels. Quantitative descriptive and between group analysis of the baseline survey were used to identify and compare sociodemographic characteristics and prioritized needs of participating communities. We conducted qualitative thematic analysis on Focus Group Discussions, using deductive main categories for triangulation: 1) prioritized needs and 2) lessons learned, with subcategories project impact, facilitators, barriers and community participation. Community participation was assessed via spidergrams. Key findings were compared with triangulation protocols.

**Results:**

A total of 348 people with disability from 6 urban settings participated in the baseline survey, with a mean age of 37.6 years (SD 23.8). Out of these, 18 participated within the four Focus Group Discussions. Less than half of the survey participants were able to read and calculate (42.0%) and reported knowledge on health care routes (46.0%). Unemployment (87.9%) and inadequate housing (57.8%) were other prioritized needs across countries. Focus Group Discussions revealed needs within health, education, livelihood, social and empowerment domains.

Participants highlighted positive project impact in work inclusion, self-esteem and ability for self-advocacy. Facilitators included individual leadership, community networks and previous reputation of participating organizations. Barriers against successful project implementation were inadequate contextualization, lack of resources and on-site support, mostly due to the COVID-19 pandemic. The overall level of community participation was high (mean score 4.0/5) with lower levels in Brazil (3.8/5) and Bolivia (3.2/5).

**Conclusion:**

People with disability still face significant needs. Community Based Inclusive Development can initiate positive changes, but adequate contextualization and on-site support should be assured.

**Supplementary Information:**

The online version contains supplementary material available at 10.1186/s12939-023-01966-8.

## Introduction

According to the “World Report On Disability” around 15% of the world’s population are people with disability (PWD) [1]. Considering Latin America, these are approximately 85 million people [2]. The United Nations “Convention on the Rights of Persons with Disabilities” acknowledges and protects human rights of PWD [3]. Although all Latin American countries have ratified this convention, PWD still face stigma, discrimination, and social disadvantages with limited access to health care, education and livelihood options. PWD and their families in Latin America are more at risk to face poverty than families without PWD [4–7]. Little research has however been done on the specific needs of PWD in Latin America, resulting in the development of rehabilitation policies and programs that do often not meet their real needs and priorities [6, 8].

Community-Based-Inclusive-Development (CBID) interventions aim to achieve a fully inclusive community [9], engaging PWD from the beginning, choosing prioritized needs and activities from divers sectors. CBID interventions are informed by the intersectoral bottom-up strategy of Community-Based Rehabilitation (CBR), developed by the World Health Organization, together with the International Labor Organization and the United Nations Educational, Scientific and Cultural Organization. The CBR matrix comprises of the five domains: 1) health, 2) education, 3) livelihood, 4) social and 5) empowerment [10]. Community participation (CP) is a key feature of CBID interventions and PWD should be involved in all stages of a program [10]. The existing evidence however suggests that CP in health interventions is often restricted to engage communities during the implementation phase of a project, e.g. to increase coverage or to reduce costs [11]. Projects involving the community in several project phases are scarce and the amount of participation seems often restrained [11]. Perceptions of PWD in Colombia on rehabilitation programs for instance indicated an unsatisfactory level of CP [6]. A high level of CP, especially during the planning of intervention components is important to assure alignment to the communities’ priorities and for sustainability of project outcomes beyond the end of intervention [12].

Implementation science seeks to identify contextual facilitators and barriers influencing the uptake of health interventions into routine use [13]. Although CBID interventions are widely used within international cooperation, there is little scientific evidence from the perspective of participating PWD. There is a general lack on operational research and especially mixed methods approaches accompanying CBID projects and the implementation of cross-country projects in particular [14, 15]. There is also a need to further assess CP in CBID programs [16], and especially in cross-country projects, where it has, to our knowledge, never been examined [17]. Being a complex process, CP significantly depends on contextual and cultural factors. Approaches considering the beneficiaries’ perspective can contribute to understand this process, perceived levels of participation and its consequences [18–20]. The participants’ perspective could moreover contribute to identify barriers and facilitators to successful project implementation [21, 22].

We retrospectively analyzed the baseline data of a CBID project conducted from 2018–2020 in Colombia, Brazil and Bolivia and combined it with prospectively collected data sources to draw lessons learned for future projects and to contribute to the scarce literature on implementation science on CBID in Latin America. The aim of the study was to identify prioritized needs of PWD in different Latin America communities, as well as lessons learned for successful project implementation.

## Methods

### Study design

**Fig. 1:**
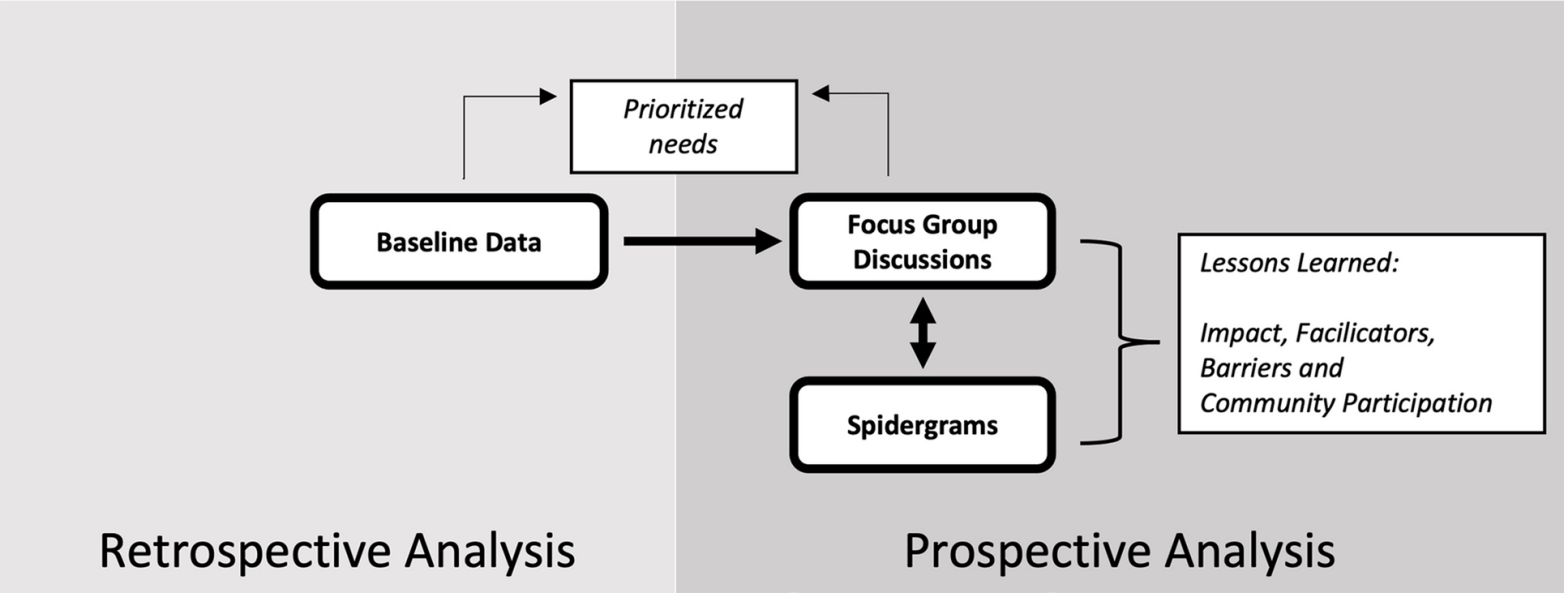
Flowchart on process of analysis and triangulation of different data sources

The study used a sequential explanatory mixed methods design [23], consisting of retrospective (baseline survey) and prospective (FGD combined with spidergrams for assessment of CP level) data sources (Fig. 1).

Key findings of different data sources were triangulated in alignment to the research objectives 1) prioritized needs and 2) lessons learned (including project impact, facilitator, barriers and level of CP). Furthermore, community profiles served as an intermediate step of analysis for local contextualization of the findings, and to identify relevant themes for further analysis.

### Study setting

**Fig. 2:**
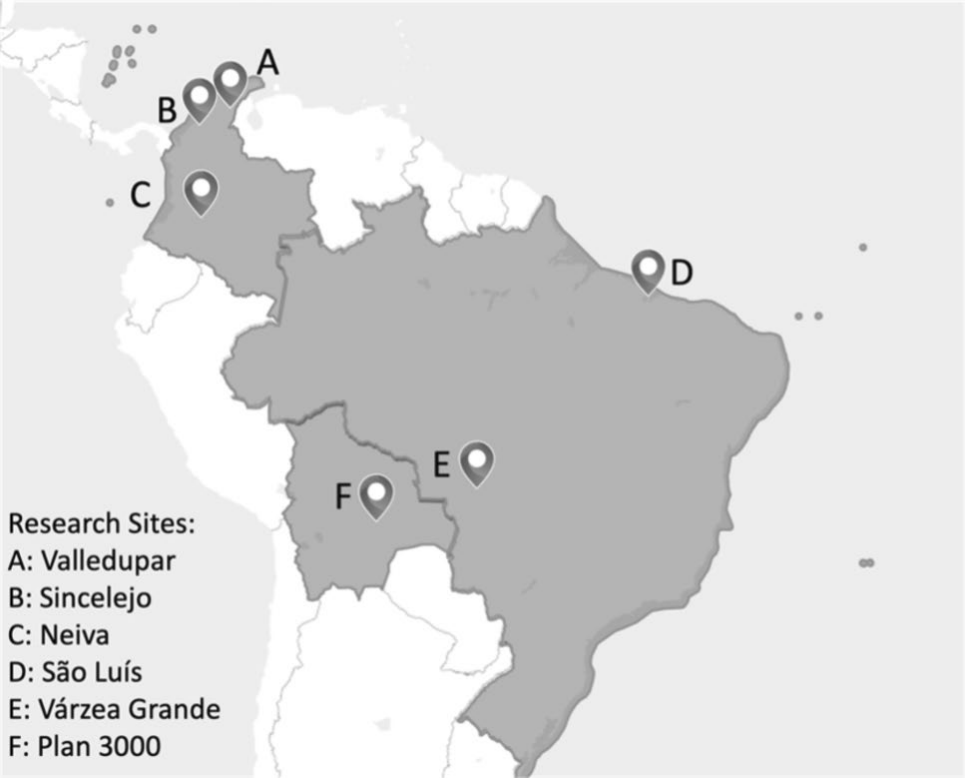
Research sites

The study was located across six urban sites in Colombia, Brazil and Bolivia (Fig. 2), that had taken part in a cross-country CBID project for PWD from 2018–2020. The project had been coordinated by the Colombian GLRA *German Leprosy and Tuberculosis Relief Association* together with Colombian leaders of the national federation of people affected by Leprosy (FELEHANSEN). Locally hired social workers had supported the implementation in Bolivia and Brazil.

### Study sample

The baseline survey was filled out by all project participants. PWD with all kinds of disabilities and their caretaker were able enroll within the project, if they were permanent residents in one of the six project communities. FGD were conducted in four projects’ sites (two in Colombia, one in Brazil, one in Bolivia to cover all countries) and convenience sampling was applied, due to difficulties arising during the pandemic (e.g., difficulties to travel and to gather in groups, COVID-19 illness and death of project participants and team members). To gain insights from different viewpoints PWD, caretakers and community leaders were eligible to participate in FGD.

### Data source description

#### Baseline survey

Anonymized data of the project baseline survey was provided by local project staff. This survey had been carried out by GLRA staff from February to June in Colombia 2018, from June to October 2018 in Bolivia and in Brazil from March to June 2019. Before the survey was administered, PWD were informed about the project and had to sign an informed consent document. When children participated in the project, informed consent was signed by their parents. The questionnaire was based on the CBR matrix, to identify local needs and priorities for the project (Additional file 1). The survey was completed on paper, either by the PWD or their caretakers. Data had been entered using Microsoft Excel Version 16. The original baseline assessment was not anonymous, because it served to register project participants, their needs and wishes to jointly plan interventions. However, the database transferred to the research team only contained data in aggregated form and no variables that would allow individual tracing of identities.

#### Focus group discussion and spidergrams

Planning of prospective data collection took place in 2021, when several project communities had been hit hard by the pandemic. FGD were held via the platform zoom® hosted at the University of Würzburg, Germany. Due to the lack of own access to internet of several participants, FGD participation was allowed from individual computers or at a meeting site. Discussions were conducted by two authors in Spanish and Portuguese (AB, SP) using a semi-structured interview guideline (Additional file 2).

Prior to FGDs, a short survey was filled out by each participant (Additional file 3), containing basic sociodemographic information and questions related to the perceived level of CP in five different project areas: needs assessment, leadership, organization, resource mobilization and management (for a detailed definition of indicators see Additional file 4). The level of CP was then rated on a continuum from 1 (narrow CP) to 5 (wide CP) for each area on spidergrams in analogy to the methodology described by Rifkin et al.) [20, 24]. Spidergrams allow to compare CP perception of different participants in the same program [20], which makes it interesting for the cross-country setting.

### Quantitative variables

Quantitative variables of interest were sociodemographic variables, such as age, gender, type of disability and baseline needs aligned to the CBR matrix, such as access to health care, educational needs or housing and working conditions.

### Qualitative dimension

The main categories and sub-categories were defined deductively, in line with the research objective to facilitate triangulation with quantitative data. Predefined main categories of interest were *prioritized needs*, with subcategories aligned to the CBR-matrix domains namely 1) health needs, 2) educational needs 3) livelihood needs 4) social needs and 5) empowerment needs [25] and *lessons learned*, with the subcategories impact of the project, facilitators and barriers of successful project implementation and community participation. Within these categories we inductively identified emerging themes.

### Analysis

Descriptive analysis on sociodemographic variables and needs was performed using SPSS Statistics 26. Differences between communities were assessed using Chi-Square or Monte Carlo Simulation, where appropriate. A p-Value of < 0.05 was considered as statistically significant [26].

FGD were recorded and transcribed verbatim. Subsequently, a structured thematic analysis was conducted in MAXQDA 2020 with a deductive-inductive approach using predefined categories aligned to the research questions as described above. Emerging themes were discussed between AB (medical student) and SP (MD, MScIH with experience in mixed methods research) who independently coded the interviews into the final coding structure using consensual coding.

Individual ratings of spidergrams were explored. A mean value was then calculated per FGD -per indicator, thus creating community-specific spidergrams (Additional file 4).

Key findings on 1) prioritized needs and 2) level of community participation emerging from the different data sources were listed within a triangulation protocol (Additional file 5). Two authors (SP and AB) independently rated these findings for agreement, partial agreement, dissonance or silence across the different data sources. The other lessons learned were derived exclusively from the FGDs (Fig. 1).

## Results

### Sociodemographic characteristics of baseline survey participants and FGD participants

A total of 348 PWD, aged 2–92 years (mean 37.6, SD 23,7) with a balanced gender distribution (48.6% female) participated in the survey (see Table 1) and (Additional file 6 for between-community-differences). Participants from Colombian municipalities made up the highest share (66.1%), followed by Brazil (23.3%). Most participants reported physical disability (34.8%), while mental disability was common in Columbian and Bolivian, but not Brazilian communities (*P* < 0.001).

**Table 1:** Sociodemographic characteristics. Participants of the baseline survey

Variable	Category	Total N (%)
**Age**	< = 15	75 (21,6)
	16–30	88 (25,3)
	31–45	54 (15,5)
	45–60	55 (15,8)
	> = 61	70 (20,1)
**Sex**	Male	179 (51,4)
	Female	169 (48,6)
**Country**	Colombia	230 (66,1)
	Bolivia	34(9,8)
	Brazil	81 (23,3)
**Category of disability**	Physical	121 (34,8)
	Mental	89 (25,6)
	Auditive	15 (4.3)
	Visual	19 (5,5)
	Other	79 (22,7)

^*^Missing values N (%): Country 3 (0,9), Category of disability 25 (7,2)

A total of four FGDs were held with 3–7 participants from October 2021 to January 2022 in the communities of Plan 3000 (Bolivia), Várzea Grande (Brazil), Neiva and Valledupar (both Colombia). FGDs lasted between 60 and 84 min. The four FGDs comprised a total of 18 participants with a majority being female participants (72.2%) and PWD (61.1%). A total of six community leaders participated in the FGD (see Table 2 for further information).

**Table 2:** Sociodemographic characteristics. Participants of the focus group discussions

Variable	Category	Total N (%)
**Age**	18–30	5 (27,8)
	31–40	3 (16,7)
	41–50	4 (22,2)
	51–60	2 (11,1)
	61–70	3 (16,7)
	> = 71	0 (0,0)
**Sex**	Female	13 (72,2)
	Male	5 (27,8)
**Community (Country)**	Plan 3000 (Bolivia)	5 (27,8)
	Várzea Grande (Brazil)	3 (16,7)
	Neiva (Colombia)	4 (22,2)
	Valledupar (Colombia)	6 (33,3)
**Category of disability**	Physical	11 (61,1)
	Mental	5 (27,8)
	Auditive	0 (0,00)
	Visual	0 (0,00)
	Other	0 (0,00)
**Status**	PWD	11 (61,1)
	Caretaker	5 (27,8)
**Leader**	Yes	6 (33,3)
	No	10 (55,6)

^*^Missing values N (%): Age 1(5,6), Category of disability 2(11,1), Status 2(11,1), Leader 2(11,1)

### Prioritized needs – Integrated results from the baseline survey and FGDs

Prioritized needs were identified within all 5 CBR-matrix domains (Table 3) [10].

**Table 3:** Side by side display of prioritized needs from the baseline survey and FGDs

*Baseline Survey*			*Focus Group Discussions*
***Variable***	***Category***	***Total N (%)***	*Themes*
***1) Health care needs***			
Cause of Disability	Congenital	152 (43,7)	-Access to assistive devices -Little access to qualified and specialized health care -Wish for specialized health care center providing free care for PWD in Bolivia
	Disease	113 (32,5)	
	Accident	45 (12,9)	
	Other	19 (5,5)	
In need of treatment	^a^	275 (79,0)	
Having access to health system		302 (86,8)	
With knowledge about health-care route		160 (46,0)	
With Knowledge about how to handle their disability		109 (31,3)	
***2) Educational needs***			
No school education		111 (31,9)	-Limited access to adequate education or trainings
Ability to read and calculate		146 (42,0)	
***3) Livelihood needs***			
Not working at the moment		306 (87,9)	-More financial and social protection -Support in employment sector
With desire for work		145 (41,7)	
Housing is not adequate for PWD		201 (57,8)	
Without access to water and/or light		13 (3,7)	
***4) Social needs***			
Vulnerable Group ^b^		148 (42,5)	-Reduce discrimination -Less institutional arbitrariness -More consideration of caretaker needs
With knowledge about rights and duties of PWD		99 (28,4)	
***5) Empowerment needs***			
Having already participated in social inclusion programs		77 (22,1)	-Need for direction in life -Desire for self-help
***Aggravated needs due to COVID-19***			
			-Loss of employment and small entrepreneurships -Deterioration of health care access -Death and illness of project staff and participants
*Example Verbatim—FGD, Plan 3000:* *“The government passed a law so that people with disabilities don`t pay anything on public transportation buses. But that`s a lie! Nobody complies with it! They don't enforce it.“*			

^a^category is “yes” if not otherwise defined

^b^Defined as Afro-Americans, Indigenous People, Displaced People

^c^Missing values N (%):Cause of disability 19 (5,5), In need of treatment 12 (3,4), Having access to health system 11 (3,2), With knowledge about health care route 22 (6,3), With knowledge about how to handle their disability 21 (6,0), No school education 66 (19), Ability to read or calculate 16 (4,6), Not working at the moment 18 (5,2), With desire for work 102 (29), Housing is not adequate for PWD 25 (7,2), Without access to water and/or light 27 (7,8), Vulnerable Group 13 (3,7), With knowledge about rights and duties of PWD 10 (2,9), Having already participated in social inclusion programs 21 (6,0)

In the domain of *1) Health care needs*, structural health care needs such as barriers to technical assistance (V1, see Verbatims in Additional file 7) or limited access to general and specialized health services—e.g. due to a lack of medical staff—were broadly discussed in FGDs (V2). In Bolivia, several participants highlighted the inexistence of free-of-charge services despite existing laws and expressed the desire for a specific health center for PWD to improve access to health care (V3). Results of the baseline survey revealed that most participants needed treatment (79.0%), whereas only 86,8% had access to health care, or knowledge on health care routes (46.0%), with much lower percentages in Bolivian participants (58,3% and 13.9% respectively; *P* < 0.001). Only about a third of the participants reported knowledge on how to handle their disability (31.3%).

2*) Educational and 3) Livelihood needs* in FGDs revealed problems in access to school inclusion, more support in the employment sector, but also the desire for more economic protection (V4-6). According to the baseline survey, the majority of PWD was not able to read and calculate (58.0%) and not working (87.9%). A considerable number of the participants were without any school education (31,9%).

4) *Prioritized needs in the social domain* emerging from FGDs referred to experiences of discrimination and institutional insensitivity and arbitrariness despite of existing rules to protect PWD (V7-8). Participants emphasized that provided help should respect not just the needs of PWD but also caretakers needs (V9). Besides living with a disability, 42.5% of the baseline survey participants belonged to another vulnerable group e.g., indigenous or displaced people. Less than a third of PWD reported knowledge about their rights and duties (28.4%).

5) Referring to the domain of *Empowerment*, FGD participants emphasized their need for a perspective in life (V10) and that provided help should focus on capacitating PWD to help themselves (V11). Some of the baseline survey participants (22.1%) had already participated in an inclusion program. FGD moreover showed that many needs were aggravated through the COVID-19 pandemic affecting specifically the labor sector (V12) and increasing the health burden faced by PWD (V13).

### Lessons learned – Integrated results from FGDs and spidergrams

To derive lesions learned from the CBID-project the results of the FGDs are presented according to the four sub-categories *1) impact of the project 2) facilitators for successful project implementation 3) barriers against successful project implementation* and *4) level of CP*. Lessons learned on CP were triangulated with results from spidergrams (Additional file 5).

1) *Impact of the project* was frequently described on participants working lives. Work inclusion was achieved for both, the caregivers (by making their daily lives easier (V14)) and PWD, by directly providing them with a job (V15) or by empowering and motivating them to start their own business or organization (V16). Participants reported that the project increased their ability for self-advocacy through knowledge generation (V17), e.g., when interacting with institutions (V18). Several participants highlighted a positively changed self-perception including increased self-esteem, that at times even motivated PWD to become role-models and change agents for other PWD (V19). Impact on self-perception was also reflected in newly received self-awareness of being able to change things on an individual (V20) but also on a community level (V21) and a sense of belonging that was described (V22). Participants perceived themselves as deserving support and inclusion projects (V23), but also reflected on their role as privileged (V24) and expressed their wish to become multiplicators and help others (V25). In contrast, Brazilian leaders expressed that the project had little impact on participants´ lives naming barriers listed below (V26). Further themes are summarized in Table 4.

**Table 4:** Lessons learned

**1) Impact of the project**	
Improvements in work inclusion and social protection Self-advocacy through knowledge generation Creation of positive self-perception Controversial reflections on project impact in Brazil Looking at an optimistic future full of plans and dreams Exchange of cultures and ideas Technical and logistic aid delivery adapted to individual needs Infrastructure improvement	
*Example Verbatim – FGD, Várzea Grande (Brazil*) *“This motivated me to try to start to study in college, to try to take that idea of empowerment and apply it in everyday life. So much so that I started my own business and even more tried to lobby for small attitude changes in my neighborhood. Small changes in your communities can make a difference”*	
**2) Facilitators for successful project implementation**	
Project coordinator and leaders as role model Supportive community networks Previous reputation of organization Expressions of resilience	
*Example verbatim—FGD, Várzea Grande* *“When you are part of GLRA and XXX*^*a*^ *said something, everybody had enormous respect (…) because GLRA is a NGO that it respects in our state. It is respected”*	
**3) Barriers against successful project implementation**	
Insufficient contextualization Good ideas but insufficient resources, time and local support to put them into practice Dealing with pride, representativeness and the risk of undermining autonomy Projects dependence on individuals Insufficient measures to assure sustainability during the pandemic Too little international exchange	
*Example Verbatim – FGD, Plan 3000* *“We agreed that there was a plan to help everyone, right? But, the xxx*^*a*^ *got sick, could no longer attend, we could no longer hold the meetings and we could not help other people either”*	
**4) Level of Community Participation**	
*Needs Assessment.*:	
Insufficient contextualization in Brazil Important role of community	Spidergramscore: 4,04
*Leadership*	
Broader inclusion of community members demanded Restrictions for entering the program	Spidergramscore: 4,09
*Organization*	
Successful integration of leader of displacement in Valledupar Active involvement of PWD is demanded	Spidergramscore: 4,10
*Resource Mobilization*	
Risk of undermining autonomy Requirement for active involvement of PWD	Spidergramscore: 3,75
*Management*	
High involvement of community in decision making in Colombia Too Little integration of participants in Decision Making in Brazil Insufficient onsite support in non-Columbian communities	Spidergramscore: 4,20
*Example Verbatim – FGD, Valledupar* *“Our leader belongs to the displacement community and well, in a way she had more knowledge of the people in need. She made a list of the people she knew and passed it on to the association—to the project coordinator, so that she could see the needs of her community.”*	

^a^*Name and function omitted for privacy*

*2) Facilitators for successful project implementation* were charismatic local leaders and the project coordinator as important motivators that often acted as role models (V27-28). Community support and an established community network were important aspects to increase trust in the project (V29), as was the pre-established local reputation of the implementing organization (V30). Participants repeatedly expressed themselves in combative/warlike language against adverse life circumstances, but also adverse circumstances during the process of project implementation (V31-V32). Especially in the context of the COVID-19 pandemic, several participants described a high level of flexibility and adaptation (V33).

*3) Barriers against successful project implementation* were mainly raised in the non-Colombian communities. Brazilian leaders criticized too little contextualization of the project (V34) and of the project focus (V35), and that they did not feel sufficiently familiar with the methodology (V36). They were leaders previously involved in social projects for people affected by leprosy and would have preferred to continue this work (V37). They expressed fear of losing reputation if the new project fails (V38) and expectations are not met. There were, however, also contrasting viewpoints: A PWD of the same community described the project as a life changing experience and inspiration (V39). Good ideas and theories were described to face insufficient resources (V40), time (V41) and local support – as e.g. project leaders where not on site(V42)—and thus hindered practical implementations in Brazil (V43). Autonomy and representativeness from the phase of designing the project (V44) was considered important. Project success depended on individuals, making the project implementation vulnerable e.g., when coordinators got sick or left the project (V45). Lack of sustainability of the project was furthermore attributed to the COVID pandemic, which significantly complicated project implementation (V46). Participants described that there was too little international exchange (V47).

**Fig. 3:**
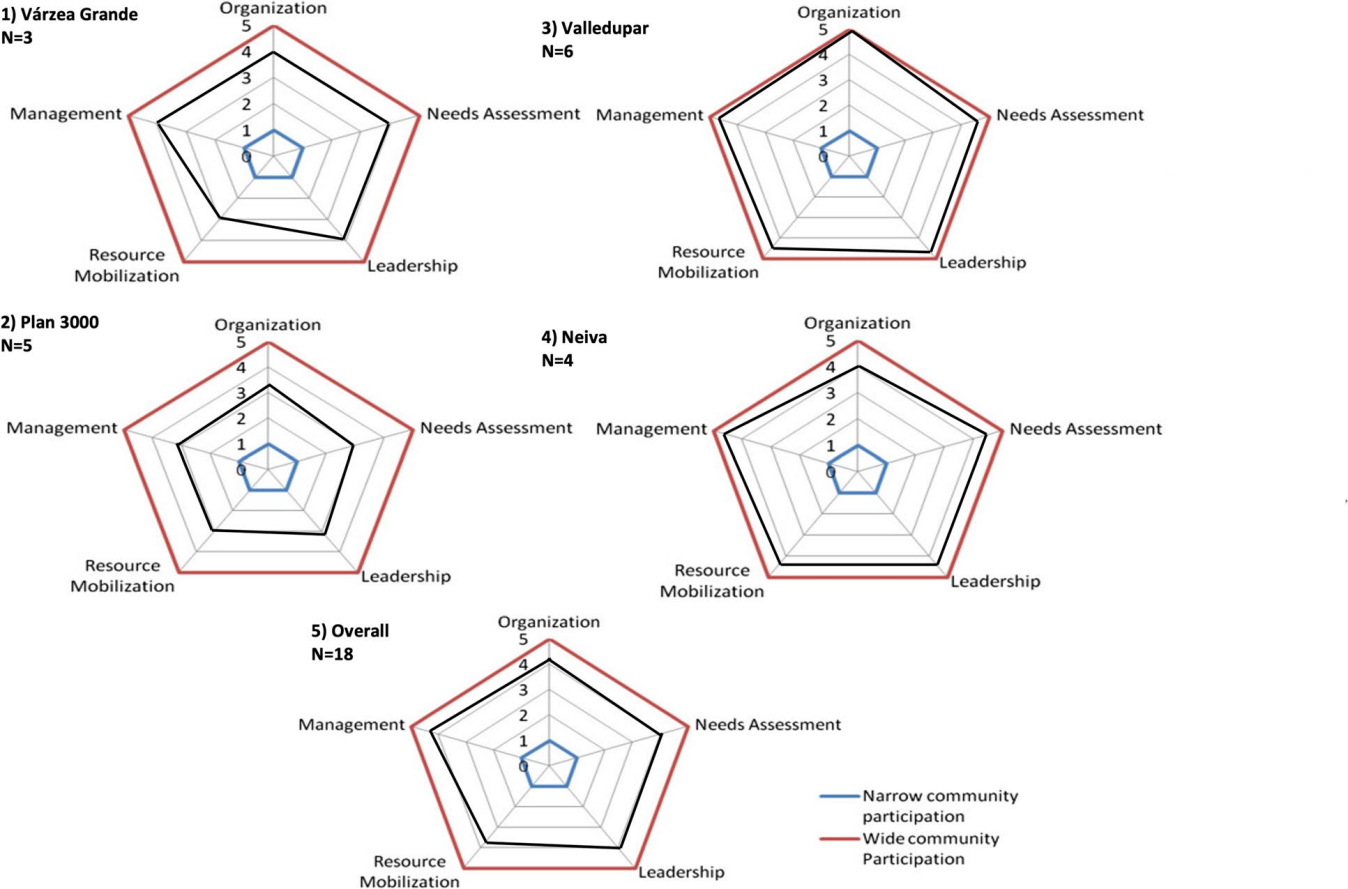
Spidergrams for community participation– adapted from Rifkin et al. [20]

*4) Level of community participation:* Overall, FGD participants considered the project to be highly participatory, with a mean score of 4.04 (out of 1–5 points). However, the non-Colombian communities indicated lower participation in the various project domains (Fig. 3; Additional file 4 for further details). The following themes were discussed within FGDs in relation to the 5 spidergram indicators (for the mean spidergram score per indicator please refer to Table 4):

*Needs assessment:* Brazilian participants described the lack of local project coordinators, too little contextualization and little autonomy in the phase of designing the project (V34-39, V44) restricting participation during the needs assessment. Colombian participants expressed the important role of community leader and the community itself during the process of need identification (V48).

*Leadership:* Although broad inclusion was attempted, the access was at times considered restrictive and participants would have wished for a broader inclusion of further community member (V49-V50).

*Organization:* Integration of existing structures was partly successful as described in Valledupar (V51). Other communities did not specifically discuss this topic.

*Resource mobilization:* Participants stated that the projects needed the active involvement of PWD, and leaders emphasized their effort to make projects accessible at a low level to achieve self-empowerment of PWD (V52). One participant gave the example that a project participant refused help because it did not involve his own resources (while re-adapting his housing conditions) and thus undermined his autonomy (V53). Across all domains, participation in this spidergram domain was rated to be the lowest, again with lower participation rates in the non-Colombian communities.

*Management:* A Colombian community reported high community involvement of PWD with a democratic way to take decision within the community (V54), while in other communities,participants stated PWD should be integrated better in decision making (V55) and more local coordinators would be needed (V42).

## Discussion

We conducted a mixed methods study consisting of the baseline survey and FGDs among participants of a cross country CBID project of six Southern American communities. Our results highlight prioritized needs of PWD around educational, labor and health care inclusion, social inclusion and the empowerment process. The CBID project initiated significant changes in the life of several participants regarding work inclusion, increased self-esteem and ability for self-advocacy, motivating some participants to become role models and multiplicators. Facilitators of successful project implementation were individual leadership, community support, previous reputation of participating organizations and resilience skills of participants, while inadequate contextualization, lack of resources and regional support were identified as barriers. Although the overall level of participation was high, we identified some challenges that should be considered when initiating transnational participatory CBID projects.

### Prioritized needs

Our study showed wide-ranging basic needs and inequities still faced by PWD, such as illiteracy, unemployment and poverty, despite the ratification of the “Convention on the Rights of Persons with Disabilities”. Data from Colombia [5, 6] suggest insufficient translation into practice of legal steps such as the Disability Law from 2013 or the National Disability Public Policy Plan [6, 27, 28]. Bolivian participants in our study moreover reported inaccessibility of free of charge services despite existing laws and programs such as universal health coverage introduced in 2019 [29]. Our results support that unequal access to services, especially to health services, for PWD goes beyond mere physical barriers and is accompanied by false assumptions, discrimination, and political exclusion [30, 31]. Reports of unfounded denial of institutional services as described by our participants as well as infrastructural barriers in public transport have previously been described in Colombia [6, 32]. Insufficient access to Brazilian hospitals for PWD is known as a relevant barrier and corresponding calls for adjustments have been made [33]. Bolivian participants in our study called for specialized hospitals for their needs. Adequate education and training of the population and specialized staff might serve as an approach to create a paradigm shift so that professionals become facilitators instead of barriers [6, 12].

Empowerment, as a central element of the CBID approach influencing all other domains [10, 17] was identified as an important topic among all data sources. As defined by the World Bank, empowerment is "increasing one's authority and control over the resources and decisions that affect one's life" [34]. The process of empowerment contributes to the identification and fulfillments of needs through facilitating rights and responsibilities of PWD, self-advocacy groups and CBID [1, 35].

### Lessons learned

Although the project was too short (and partly interrupted by the pandemic) to measure sustainable impact, several initial changes were reported. In line with a project from India, we found multidimensional positive impact on the lives of PWD and their families/caretakers [36]. Positive changes were reported in most of the domains of the CBR matrix, i.e., health, livelihood, education, social participation, with the aspect of work inclusion playing a key role. Although access to employment through CBID programs has been poorly studied [16, 36], there is evidence that interventions providing socioeconomic support through self-employment are beneficial on self-esteem, sense of autonomy and empowerment [37]. Labor inclusion can moreover decrease stigma and change attitudes towards PWD. Inequity within the labor sector is mostly caused by negative attitudes towards PWD hampering access to jobs—particularly of women- and not related to productivity or aspects of human capital [36, 38, 39]. Narratives of participants indicated that changes around self-esteem and empowerment were often achieved. Several participants described their plans of starting own businesses, progressing in education, or felt more empowered to stand up against discrimination. This change in life often led to plans of becoming a leader and multiplicator to empower other PWDs. This goes in line with the experience made by GLRA in Colombia among people affected by leprosy, who now helped to plan and implement the cross-country project to multiply their own previous experience. Similar effects are described in community movements, such as self-help groups [40].

Participants, especially in Brazil, however also mentioned a lack of sustainability and unmet expectations. While this was partly explained by the severe restrictions and illness of project staff during the pandemic, some barriers should be considered when planning cross-country CBID projects:

The Brazilian communities were formerly involved in other GLRA projects with a narrower focus on income generating activities and Leprosy control and would have liked these projects to be continued. The background of participating communities and existing local structures should therefore already be considered during the initial planning phase of the project to assure ownership. Simultaneously, it is essential for multi-site projects to find the balance between fidelity and local adaptations to achieve contextualization [21, 41]. Reduced CP across all project phases can lead to reduced sustainability of a program [19].

In line with findings from Belize [37], we identified the essential role of project coordinators and leaders as role-models and multipliers being able to facilitate processes and determine success of a project. At the same time, this key role is also an Achilles' heel of projects, since if local coordinators leave or become ill, a project can falter. Partly due to the pandemic the non-Colombians lacked a continued onsite support from Colombia, which made it harder to put the CBID methodology, that was perceived as not very pragmatic and hard to understand for some leaders into practice. Regarding CP some participants noted that the integration of even more PWD would be desirable. Although CBID measures are considered cost-effective, a balance must be struck between the broadest possible integration of PWD and scarce resources [10]. Working closely in the community can certainly be helpful here.

The COVID-19 pandemic has aggravated some of the participating PWD's problems and added new ones. PWDs are more vulnerable to COVID-19 and the consequences of the measures taken to combat the pandemic [42, 43]. In South America, government action during the COVID pandemic was partly in place, but also inconsistent or insufficiently inclusive leading to a high burden, e.g., in countries such as Brazil [44]. While the pandemic severely affected the lives of our participants and the implementation of the project, some highlighted their increased resilience and flexibility against these and other adverse conditions after the project. These resilience characteristics can be addressed during interventions [45].

## Limitations

Our study has some limitations. The baseline assessment was not originally collected for scientific purposes. Therefore, some inconsistencies may exist (e.g., the lack of possibility for multiple responses). Moreover, the sampling for both quantitative and qualitative data was by convenience, including PWD that wanted to participate in the project and FGDs. Their priorities and views might not represent other PWD from their communities and are not transferable to other contexts. Data collection for our study took place during the COVID-19 pandemic, so the German study-team could not work with colleagues on the ground in the researched areas and exchange was restricted only to digital platforms.

## Conclusion

Our study supports that there are still major inequalities in the translation of the “Convention on the Rights of Persons with Disabilities” into practice. Prioritized needs emerged among several domains, such as social participation and empowerment as well as inadequate access to health, education, and employment systems, especially in times during and after the COVID pandemic. These needs of PWD are still insufficiently covered in various community settings in Latin America. Our lessons learned provide support that CBID interventions can initiate positive changes in PWD lives. However, several aspects should be considered during their implementation including adequate contextualization, the important role of the project coordinator and high levels of CP.

### Supplementary Information

**Additional file 1.** 

**Additional file 2.** 

**Additional file 3.** 

**Additional file 4.** 

**Additional file 5.** 

**Additional file 6.** 

**Additional file 7.**

## Data Availability

If you have a reasonable request for the analyzed data, please contact the authors.

## Abbreviations

PWD: People With Disability
CBID: Community-Based-Inclusive-Development
CBR: Community Based Rehabilitation
CP: Community Participation
GLRA: German Leprosy and Tuberculosis Relief Association
PWD: Person with disability
WHO: World Health Organization
