# Supplementary material for: Implementing community based inclusive development for people with disability in Latin America: a mixed methods perspective on prioritized needs and lessons learned
Source: Int J Equity Health. 2023 Aug 4;22:147. doi: 10.1186/s12939-023-01966-8 (PMC10403844; doi:10.1186/s12939-023-01966-8)
Supplement: Supplementary file 1 — Additional file 1. [file 12939_2023_1966_MOESM1_ESM.docx]

# **Additional File 1: Baseline survey**

# **Original Spanish Version**
