# Supplementary material for: Implementing community based inclusive development for people with disability in Latin America: a mixed methods perspective on prioritized needs and lessons learned
Source: Int J Equity Health. 2023 Aug 4;22:147. doi: 10.1186/s12939-023-01966-8 (PMC10403844; doi:10.1186/s12939-023-01966-8)

# **Additional File 2: Semistructured Interview Guide for Focus Group Discussions (FGD)**

*Community-Based-Inclusive-Development (CBID) in a cross-country context: Experiences of community members participating in a project for People with Disability (PWD) in Colombia, Bolivia and Brazil - A mixed-methods study*

(This document will be translated to Spanish and Portuguese before conducting the FGD)

| Date of the  interview: |  |
| --- | --- |
|  |  |
| Duration of the conversation: |  |
|  |  |
| Name of  interviewers: |  |
|  |  |
| Interview  number: |  |
|  |  |
| Participants Pseudonyms: |  |
|  |  |

## Introduction:

1. Welcoming words and technical "check-in
   1. Each participant should activate his microphone once and speak a few words to check the sound quality and the quality of internet connection.
2. Introduction of the interviewers
3. Explanation about the goals of this discussion and about the timeframe of this session
   1. “All of you have been involved in the social inclusion project of the DAHW. In this discussion, we want to find out how you experienced the project and especially how involved you were in the project. We also want you to discuss how the project did influence your daily lives. So that is why we gathered today, and I am glad to have the chance to ask you a few questions about these themes”
4. Information about conditions of participation and data protection (analogous to informed consent document)
5. Information about „Zoom behavior rules”
   1. “Cameras are switched of”
   2. “Microphones are only activated when you want to say something.”
   3. “Before each speech, briefly state your pseudonym. If someone does not remember his or her pseudonym, he or she can still call in now.”
6. Switching on the recording device and recording function of ZOOM

## Interview questions:

“I would now like to ask you questions about your experiences in the project. Please take your time and feel free to say anything that comes to your mind. You are welcome to respond to statements made by other participants, to contradict or reinforce statements so that a discussion can take place.”

| Topic | Question | Potential further questions |
| --- | --- | --- |
| Experience of the project | “So for the beginning, I would like you to discuss openly about the project. Can you please describe what the project was like?” | “How did you all experience being involved in the project?” |
| Lessons Learned (Impact, Facilitators, Barriers) | Can you please tell us if your experienced any changes (in your life) during or after the project? Please tell as anything that comes to mind. | “Were there any influences on your health/work life/education/way of living/self-perception?”  “Did you experience changes at the  Community level? “  “What caused these changes?”  “What helped you during the project/What did you like about the project?”  “What did you not like about the project?”  “Were there any negative changes?” and “What caused these Changes?”  “Where your expectations met?” and “Why not?”  “What would have had to be different so that you would have benefited more?” |
| International context | “How did you experience the international context?” | “Were there any benefits from the international context?”  “Were there any difficulties due to the international context ?” |
| Participation | Thank you so far, as a last step in this discussion we want to explore the topic of participation more deeply. Could you please tell everything that comes to mind? | “Where would you have liked to see more participation in the project? “  These questions should only be used when there is need to clarify the meaning of the indicators:    **1. Need assessment**  “What was the community’s role like in designing the program? “  “How was the community involved to articulate its needs?”  **2. Leadership**  “How was the community’s involvement as a decision-maker in the project? “  **3. Organization**  “How were preexisting organizations and structures involved in the project?”  **4. Resource Mobilization**  “How was the community able to contribute their own resources to the project?”  **5. Management**  “To what extent was the community able to decide how the project would develop?” |

| Useful props:  You have said that […]. Can you give us a concrete example?  Can you tell me more about it?  We have now heard a lot from […], I would like now the […] to give their opinion.  Thank you. What do other people think?  That was a very impressive and interesting statement, in order to stay a little closer to the topic, but I would like to know from you […]?  You have said that: […]. Did I understand that correctly? |
| --- |

“That is all I wanted to discuss with you for now. Is there anything else you would like to add? Something that has perhaps not been mentioned so far.”

“Thank you very much for participating in this study and I wish you all the best for the future. I want you to quickly stay so that we can finish collecting your sociodemographic data.”

## Post FGD Checklist

Verification of the recording/ Safely saved? Cover sheet completed? Demographic data complete?
Writing down important impressions/ Emerging Themes

## Post FGD Checklist

| Verification of the recording/ Safely saved? Cover sheet completely filled in? |  |
| --- | --- |
| Personal data complete? |  |
| Writing down important impressions/emerging themes |  |
|  |  |

## Reflections:

How did the interview go, how did I feel? Did I feel like I was influencing the answers at certain points? Did I talk about myself? Were there any comprehension problems? Were there moments/causes when the flow of speech got bogged down? Please include any other notes that might be relevant

Further reflections

What research objectives/questions were addressed? Are there topics that are relatively saturated?

Emerging themes:

Are there topics that should be explored more in other interviews?


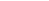

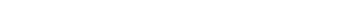

Supplement: Supplementary file 2 — Additional file 2. [file 12939_2023_1966_MOESM2_ESM.docx]
