# Supplementary material for: Implementing community based inclusive development for people with disability in Latin America: a mixed methods perspective on prioritized needs and lessons learned
Source: Int J Equity Health. 2023 Aug 4;22:147. doi: 10.1186/s12939-023-01966-8 (PMC10403844; doi:10.1186/s12939-023-01966-8)
Supplement: Supplementary file 3 — Additional file 3. [file 12939_2023_1966_MOESM3_ESM.docx]

# **Additional File 3: Questionnaire on Community Participation**

1. **Original Version in Spanish**

#### Seudónimo:

#### Por favor elija un seudónimo que pueda recordar bien (escríbalo de nuevo en un papel si es necesario). Sólo nos dirigiremos a usted con un seudónimo en las discusiones de los grupos focales.

#### Género:

- hombre
- mujer
- diverso

#### Estado:

- Persona con discapacidad
- Cuidador

#### Categoría de discapacidad de uno mismo o de la persona cuidada:

- Físico
- Visual
- Auditivo
- Cognitivo
- Otros

#### Edad:

#### Región:

- Plan 3.000
- Várzea Grande
- Neiva
- Valledupar
- 18-30
- 31-40
- 41-50
- 51-60
- 61-70
- 71-80
- >80

#### Líder comunitario:

- Sí
- No

#### Nivel de participación en el proyecto

Quisieramos saber cómo perciben la participación de la comunidad en el proyecto. Por lo tanto, les pedimos que por favor clasifiquen el nivel de participación de la comunidad en cada fase del proyecto. Se trata de la participación de la comunidad en su conjunto, no de su participación individual. Seleccione una de las opciones (de muy baja a muy alta). Aquí se pueden ver las 5 fases.

**1. Evaluación de necesidades:** el papel que desempeñan los participantes en la identificación de sus necesidades y en el diseño del programa.

La participación en el área de evaluación de necesidades fue:

*muy baja - baja – intermedia - alta - muy alta*

**2. Liderazgo:** la inclusividad y representatividad de todos los grupos de interés de la comunidad.

La participación en el área de liderazgo fue:

*muy baja - baja – intermedia - alta - muy alta*

**3. Organización:** el grado de integración o colaboración del programa con las estructuras o redes comunitarias preexistentes.

La participación en el área de organización fue:

*muy baja - baja – intermedia - alta - muy alta*

**4. Movilización de recursos:** la capacidad de las comunidades para movilizar y aportar recursos al programa

La participación en el área de movilización de recursos fue:

*muy baja - baja – intermedia - alta - muy alta*

**5. Gestión:** capacidad de la comunidad para tomar decisiones sobre la dirección y el desarrollo del programa.

La participación en el área de gestión fue:

*muy baja - baja – intermedia - alta - muy alta*

*Notas (aquí hay espacio, por si quiere contarnos algo más)*:__________________________

_________________________________________________________________________________________________________________________________________________________________________________________________________________________________

___________________________________________________________________________

1. **English Translation**

#### Pseudonym:

#### Please choose a pseudonym that you can remember well (write it again on paper if necessary). We will only address you with a pseudonym during the focus group discussions.

#### Gender:

- man
- woman
- diverse

#### Disability status:

- person with disability
- caregiver

#### Disability category of oneself or the person cared for:

- Physical
- Visual
- Auditive
- Cognitive
- Others

#### Age:

#### Community/City:

- Plan 3.000
- Várzea Grande
- Neiva
- Valledupar
- 18-30
- 31-40
- 41-50
- 51-60
- 61-70
- 71-80
- >80

#### Community Leader:

- yes
- no

#### Level of participation in the project

We would like to know how you perceived the participation of the community during the project. Therefor, we ask you to please classify the level of community participation in each phase of the project. We are interested about the participation of the community as a whole, not about your individual participation. Select one of the options (from very low to very high). The 5 phases are depicted below.

**1. Needs assessment:** the role that participants play in identifying their needs and designing the program.

Participation during the phase of needs assessment was:

*Very low low intermediate high very high*

**2. Leadership:** inclusiveness and representativeness of all groups of interest from the community.

Participation in the area of leadership was:

*Very low low intermediate high very high*

**3. Organization:** the degree of integration or collaboration of the program with pre-existing community structures or networks.

Participation in the organizational area was:

*Very low low intermediate high very high*

**4. Resource mobilization:** the ability of communities to mobilize and contribute resources to the program

Participation in the resource mobilization area was:

*Very low low intermediate high very high*

**5. Management:** capacity of the community to make decisions about the direction and development of the program.

Participation in the management area was:

*Very low low intermediate high very high*

*Notes (here you have space, in case you want to tell us anything else):*

*______________________________________________________________________________________________________________________________________________________________________________________________________________________________________________________*
