# Supplementary material for: Implementing community based inclusive development for people with disability in Latin America: a mixed methods perspective on prioritized needs and lessons learned
Source: Int J Equity Health. 2023 Aug 4;22:147. doi: 10.1186/s12939-023-01966-8 (PMC10403844; doi:10.1186/s12939-023-01966-8)
Supplement: Supplementary file 4 — Additional file 4. [file 12939_2023_1966_MOESM4_ESM.docx]

**Additional File 4:**

**a) Definition of indicators for Community participation according to Rifkin and Colleagues:**

| *Indicator:* | D*efinition* |
| --- | --- |
| *Needs assessment* | The roles played by program beneficiaries in identifying their health needs and in designing the community intervention. |
| L*eadership* | The inclusiveness and representativeness of all community interests groups. |
| *Organization* | The extent to which new community interventions integrate or collaborate with pre-existing community structures or networks |
| *Resource mobilization* | The communities’ ability to mobilize and contribute resources towards a community–based intervention |
| *Management* | The communities’ capacity to take decisions about the programs’ direction and development. |

From: Baatiema, L., Skovdal, M., Rifkin, S. *et al.* Assessing participation in a community-based health planning and services programme in Ghana. *BMC Health Serv Res* **13,**233 (2013). https://doi.org/10.1186/1472-6963-13-233

b**) Spidergram-Scores**

| Region | | N | Minimum | Maximum | Std. Deviation | Mean |
| --- | --- | --- | --- | --- | --- | --- |
| Plan 3000 | PartNeedsassesment | 5 | 3 | 3 | 0,00 | 3,00 |
|  | PartLeadership | 5 | 2 | 4 | 0,84 | 3,20 |
|  | PartOrganisation | 5 | 2 | 4 | 0,89 | 3,40 |
|  | PartResourceMobil | 5 | 2 | 4 | 0,71 | 3,00 |
|  | PartManagment | 5 | 2 | 4 | 0,84 | 3,20 |
|  | Valid N (listwise) | 5 |  |  |  |  |
| Varzea Grande | PartNeedsassesment | 3 | 3 | 5 | 1,00 | 4,00 |
|  | PartLeadership | 3 | 3 | 5 | 1,00 | 4,00 |
|  | PartOrganisation | 3 | 3 | 5 | 1,00 | 4,00 |
|  | PartResourceMobil | 3 | 2 | 4 | 1,00 | 3,00 |
|  | PartManagment | 3 | 3 | 5 | 1,00 | 4,00 |
|  | Valid N (listwise) | 3 |  |  |  |  |
| Neiva | PartNeedsassesment | 4 | 4 | 5 | 0,58 | 4,50 |
|  | PartLeadership | 4 | 4 | 5 | 0,58 | 4,50 |
|  | PartOrganisation | 4 | 4 | 4 | 0,00 | 4,00 |
|  | PartResourceMobil | 4 | 4 | 5 | 0,58 | 4,50 |
|  | PartManagment | 4 | 4 | 5 | 0,50 | 4,75 |
|  | Valid N (listwise) | 4 |  |  |  |  |
| Valledupar | PartNeedsassesment | 6 | 4 | 5 | 0,52 | 4,67 |
|  | PartLeadership | 6 | 3 | 5 | 0,82 | 4,67 |
|  | PartOrganisation | 6 | 5 | 5 | 0,00 | 5,00 |
|  | PartResourceMobil | 6 | 3 | 5 | 0,84 | 4,50 |
|  | PartManagment | 6 | 4 | 5 | 0,41 | 4,83 |
|  | Valid N (listwise) | 6 |  |  |  |  |
|  |  |  |  |  |  |  |
| Overall | Need Assesment | 18 |  |  |  | 4,04 |
|  | Leadership | 18 |  |  |  | 4,09 |
|  | Organisation | 18 |  |  |  | 4,10 |
|  | Resource Mobilisation | 18 |  |  |  | 3,75 |
|  | Management | 18 |  |  |  | 4,20 |
|  |  |  |  |  |  |  |
|  | Average Overall | 18 |  |  |  | 4,04 |
|  |  |  |  |  |  |  |
