# Supplementary material for: Implementing community based inclusive development for people with disability in Latin America: a mixed methods perspective on prioritized needs and lessons learned
Source: Int J Equity Health. 2023 Aug 4;22:147. doi: 10.1186/s12939-023-01966-8 (PMC10403844; doi:10.1186/s12939-023-01966-8)
Supplement: Supplementary file 5 — Additional file 5. [file 12939_2023_1966_MOESM5_ESM.docx]

**Additional File 5: Triangulation Protocol**

**Need priorities:**

Need priorities identified in both data sources were increased accessibility and inclusion to the health, education, and labor sector. PWD still faced unmet basic and financial needs. Social participation and empowerment were found to be essential. Several key findings were consistent across data sets (agreement 6 out of 14, partial agreement 3 out of 14), the others were topics that were addressed in only one of the two data sets, such as emergence of new priorities during the project (e.g. disability health center in FGD) (Silence 5 of 14). There was no disagreement between key findings.

| ***Meta theme*** | ***Themes Baseline Data*** | ***Themes***  ***Focus Group Discussion*** | ***Convergence Assessment*** |
| --- | --- | --- | --- |
| *Health* | *Highest share of disability category: physical disability,*  *Highest share of disability cause: congenital disability*  *Majority in need of treatment.* | *Access barriers and participation restrictions due to physical disability were frequently discussed.*  *Specific treatment needs for wheelchair users, such as physiotherapy, and prevention of urinary tract infection.* | *Partial agreement* |
|  | *42% In need of technical assistance* | *Access to assistive devices as prioritized needs* | *Agreement* |
|  | *15% without access to health system, Majority without knowledge about health care route* | *Little access to qualified and specialized health care*  *Wish for specialized health care center providing free care for PWD in Bolivia* | *Agreement* |
|  | *Majority not knowing how to handle disability* |  | *Silence* |
|  |  | *Mental health needs* | *Silence* |
| *Education* | *1/3 without education*  *40% don´t know to read or calculate* | *Little access to adequate education and trainings* | *Agreement* |
| *Livelihood* | *About 90% unemployed* | *Support in employment sector* | *Agreement* |
|  | *Majority without adequate housing*  *Existence of participants without access to water and/or light* | *More social and financial protection*  *Adaptation of housing conditions during the project apparently only prioritized in few individuals* | *Partial Agreement* |
| *Social* | *Majority without knowledge about rights and duties* | *Reduce Discrimination*  *Less institutional arbitrariness* | *Agreement* |
|  | *High share of participants belonging to other vulnerable group* | *Belonging to other vulnerable groups was not discussed as major theme. Being part of a network for displaced people was mentioned as facilitator for community access.* | *Partial agreement* |
|  |  | *More consideration of caretakers needs* | *Silence* |
| *Empowerment* | *92% with desire to belong to a PWD group. 12,6% already belonging to a social group and 11% belonging to a support group* | *Need for direction in life* | *Agreement* |
|  |  | *Desire for self-help* | *Silence* |
| *Aggravated needs due to COVID-19* |  | *Loss of employment and small entrepreneurships*  *Deterioration of health care access*  *Death and illness of project staff and participants* | *Silence* |

**Community participation**

Triangulation of data sources

Both FGDs and spidergrams showed a high level of participation in Colombian communities, while CP was rated lower in Bolivia and Brazil. FGD enabled a deeper understanding of the challenges arising during the different phases of the project (Additional File 5)

| **Meta-Theme** | **Focus Group Discussion** | **Spidergrams** | **Convergence Assessment** |
| --- | --- | --- | --- |
| Need Assessment | -Insufficient contextualization in Brazil  - No explanation for individual low ratings in Bolivia | 4,04 | Partial agreement |
| Leadership | -Broader inclusion of community members demanded  -Restrictions for entering the program | 4,09 | Partial agreement |
| Organization | -Successful integration of leader of displacement in Valledupar | 4,10 | Partial agreement |
| Resource Mobilization | -Risk of undermining autonomy  -Active involvement of PWD is demanded | 3,75 | Partial agreement |
| Management | -Insufficient onsite support in Brazil and Bolivia  -High involvement of community in decision making in Colombia | 4,20 | Partial agreement |

Need Assessment -Insufficient contextualization in Brazil

- No explanation for individual low ratings in Bolivia

Leadership -Broader inclusion of community members demanded

-Restrictions for entering the program

Organization -Successful integration of leader of displacement in Valledupar

Resource Mobilization -Risk of undermining autonomy

-Active involvement of PWD is demanded

Management -Insufficient onsite support in Brazil and Bolivia

-High involvement of community in decision making in Colombia
