# Supplementary material for: Implementing community based inclusive development for people with disability in Latin America: a mixed methods perspective on prioritized needs and lessons learned
Source: Int J Equity Health. 2023 Aug 4;22:147. doi: 10.1186/s12939-023-01966-8 (PMC10403844; doi:10.1186/s12939-023-01966-8)
Supplement: Supplementary file 6 — Additional file 6. [file 12939_2023_1966_MOESM6_ESM.docx]

**Additional File 6: Between Community Differences**

| Variable | Category | Várzea Grande | São Luis | Neiva | Sincelejo | Valledupar | Plan 3000 | Total | P-Value |
| --- | --- | --- | --- | --- | --- | --- | --- | --- | --- |
|  |  | Total N (%) | Total N (%) | Total N (%) | Total N (%) | Total N (%) | Total N (%) | Total N (%) |  |
| N |  | 52 | 31 | 81 | 81 | 67 | 36 | 348 |  |
| Average Age |  | 47,35 | 52,85 | 44,33 | 24,69 | 33,15 | 32,65 | 37,6 |  |
| Age | <=15 | 4 (7,7) | 2 (6,5) | 15 (18,5) | 31 (38,3) | 13 (19,4) | 10 (27,8) | 75 (21,6) | 0,00* |
|  | 16-30 | 7 (13,5) | 2 (6,5) | 15 (18,5) | 30 (37,0) | 24 (35,8) | 10 (27,8) | 88 (25,3) | 0,00* |
|  | 31-45 | 13(25,0) | 7 (22,6) | 3 (16,0) | 8 (9,9) | 10 (14,9) | 3 (8,3) | 54 (15,5) | 0,85 |
|  | 45-60 | 7 (13,5) | 7 (22,6) | 13 (16,0) | 7 (8,6) | 14 (20,9) | 7(19,6) | 55 (15,8) | 0,23 |
|  | >=61 | 19 (36,5) | 9 (29,0) | 25 (30,9) | 5 (6,2) | 6 (9,0) | 6 (16,7) | 70 (20,1) | 0,00* |
| Sex | Male | 25 (48,1) | 12 (38,7) | 41 (50,6) | 37 (45,7) | 42 (62,7) | 22 (61,1) | 179 (51.4) | 0,15 |
|  | Female | 27 (51,9) | 19 (61,3) | 40 (49,4) | 44 (54,4) | 25 (37,3) | 14 (38,9) | 169 (48.6) |  |
| Vulnerable Group* defined as Afroamericans, Indigenous People, Displaced People) |  | 20 (38,4) | 24 (77,5) | 11 (13,5) | 46 (56,8) | 46 (68,6) | 1 (2,8) | 148 (42,5) | 0,00* |
| Category of Disability | Physical | 14 (26,9) | 9 (29,0) | 25 (30,9) | 29 (35,8) | 31 (46,3) | 13 (36,1) | 121 (34,8) | 0,36 |
|  | Mental | 1 (1,9) | 0 (0,0) | 18 (22,2) | 34 (42,0) | 26 (38,8) | 10 (27,8) | 89 (25,6) | 0,00* |
|  | Auditive | 2 (3,8) | 1 (3,2) | 8 (9,9) | 2 (2,5) | 2 (3,0) | 0 (0,0) | 15 (4,3%) | 0,09 |
|  | Visual | 5 (9,6) | 0 (0,0) | 6 (7,4) | 2,5 (2) | 2 (3,0) | 5 (11,1) | 19 (5,5) | 0,12 |
|  | Other | 22 (42,3) | 17(54,8) | 17 (21,0 | 14 (17,3) | 1 (1,5) | 8 (22,2) | 79 (22,7) | 0,00* |
| Cause of Disability | Congenital | 17 (32,7) | 8 (25,8) | 31 (38,3) | 59 (72,8) | 27 (40,3) | 10 (27,8) | 152 (43,7) | 0,00* |
|  | Disease | 16 (30,8) | 16 (51,6) | 37 (45,7) | 12 (14,8) | 15 (22,4) | 17 (47,2) | 113 (32,5) | 0,00* |
|  | Accident | 6 (11,5) | 5 (16,1) | 8 (9,9) | 6 (7,4) | 14 (20,9) | 6 (16,7) | 45 (12,9) | 0,08 |
|  | Other | 6 (11,5) | 1 (3,2) | 4 (4,9) | 4 (4,9) | 1 (1,5) | 3 (8,3) | 19 (5,5) | 0,16 |
| In need of treatment |  | 44 (84,6) | 26 (83,9) | 60 (74,1) | 54 (80,2) | 54 (80,6) | 26 (72,2) | 275 (79,0) | 0,10 |
| Having Access to health system |  | 44 (84,6) | 24 (77,4) | 74 (91,4) | 79 (97,5) | 60 (89,6) | 21 (58,3) | 302 (86,8) | 0,00* |
| With Knowledge about health-care route |  | 29 (55,8) | 21 (67,7) | 38 (46,9) | 46 (56,8) | 21 (31,3) | 5 (13,9) | 160 (46,0) | 0,00* |
| With Knowledge about how to handle their disability |  | 21 (40,4) | 6 (19,4) | 17 (21,0) | 44 (54,3) | 13 (19,4) | 8 (22,2) | 109 (31,3) | 0,00* |
| No school education |  | 2 (3,8) | 10 (32,3) | 28 (34,6) | 30 (37,0) | 26 (38,8 | 15 (41,7) | 111 (31,9) | 0,30 |
| Ability to read and calculate |  | 19 (36,5) | 12 (38,7) | 36 (44,4) | 37 (45,7) | 26 (38,8) | 16 (44,4) | 146 (42,0) | 0,99 |
| Not working at the moment |  | 43 (82,7) | 28 (90,3) | 73 (90,1) | 69 (85,2) | 58 (86,6) | 35 (97,2) | 306 (87,9) | 0,52 |
| With desire for work |  | 9 (17,3) | 8 (25,8) | 1 (50,6) | 8 (46,9) | 3 (49,3) | 16 (44,4) | 145 (41,7) | 0,00* |
| Housing is not adequate for PWD |  | 26 (50,0) | 23 (74,2) | 50 (61,7) | 39 (48,1) | 40 (59,7) | 23 (63,9) | 201 (57,8) | 0,96 |
| Without access to water and/or light |  | 0 (0.0) | 1 (3,2) | 2,5% (2) | 2 (2,5) | 7 (10,4) | 1 (2,8) | 13 (3,7) | 0,36 |
| With knowledge about rights and duties of PWD |  | 12 (23,1) | 3 (9,7) | 16 (19,8) | 52 (64,2) | 11 (16,4) | 5 (13,9) | 99 (28,4) | 0,00* |
| Having already participated in social inclusion programs |  | 10 (19,2) | 3 (9,7) | 19 (23,5) | 25 (30,9) | 8 (26,9) | 2 (5,6) | 77 (22,1) | 0,02 |
|  |  |  |  |  |  |  |  |  |  |

*****statistically significant
