# Supplementary material for: Implementing community based inclusive development for people with disability in Latin America: a mixed methods perspective on prioritized needs and lessons learned
Source: Int J Equity Health. 2023 Aug 4;22:147. doi: 10.1186/s12939-023-01966-8 (PMC10403844; doi:10.1186/s12939-023-01966-8)
Supplement: Supplementary file 7 — Additional file 7. [file 12939_2023_1966_MOESM7_ESM.docx]

**Additional File 7: Verbatims in English**

| Number | Source (referece to which community) | Speaker | Verbatim |
| --- | --- | --- | --- |
| V1 | FGD (Valledupar) | Caregiver(Valledupar)1 | It was very difficult for him to move from one place to another because the walker was damaged (…) the EPS (National health insurance) had not given him a walker either. He had already requested it, but it did not arrive. So priority was given to this basic need, because many people needed this help. |
| V2 | FGD (Várzea Grande) | PWD(VárzeaGrande)1 | I am a wheelchair user in need of a monthly urologist appointment, because of urinary infections (…) and there is only one appointment in 17 weeks. And then there is a very large group that is not able to access this at all, through the existing structures. They are very, very outdated. So, it is hard to create partnerships: someone who provides doctors, physiotherapists, psychologists and urologists. |
| V3 | FGD (Plan 300) | PWD(Plan 3000)1 and Leader(Plan3000)1 | Nothing is free-of-charge here, you have to pay for everything, everything! If you get a prescription you have to buy it yourself, if you get an appointment to see a physician, you have to pay the appointment! No, they don´t care for us free-of-charge here.  (…) if the Bolivian government could be touched so that there could be a specialized health center in each department just for (PWD), so that they can go there. Unfortunately, they have made laws that they do not comply with and if you have to go to a public hospital, right now it's terrible, difficult. |
| V4 | FGD (Neiva) | Leader(Neiva)1 | And more than that, I did this project with my mom, because she sew and she needed the machine. |
| V5 | FGD (Plan 3000) | Leader(Plan3000)2 | For the boys in this this project (the goal might be) to go and look for an institute where the boys can learn computers or they can learn to fix cell phones, as well as to fix electronics. |
| V6 | FGD (Neiva) | PWD(Neiva)1 | A little bit more than that - he makes very delicious gelatin and he's very well known for it - but he needs a little bit more than that! Yes a little bit more than that! Yes, like that. A budget to get us up again. |
| V7 | FGD (Plan 3000) | Leader(Plan3000)1 | Stigma exists, we still know that. It does exist. There are people that are good in a way, but a large majority of people feels discriminated. (…) I hope - that they work a little more on this aspect (…). For example, the government passed a law so that people with disabilities on public transportation buses do not pay anything. But lie! Nobody complies with it! They don't enforce it! Or the senior citizens who pay only half fare, they don't comply with it either. |
| V8 | FGD (Plan 3000) | Leader(Plan3000)2 | (…) unfortunately, in the health centre they are not well received, they are not well attended. Why is that? Because there are so many people, that there are supposed to be priorities. So they are almost… as if they were marginalized, there is no priority for them. |
| V9 | FGD (Valledupar) | Caregiver(Valledupar)1 | Sometimes, because we have to care for our children, we forget that we exist. But we forget, that we can also be useful, that, that time is not only for our children, but also for ourselves. |
| V10 | FGD (Várzea Grande) | Leader(VárzeaGrande1) | We had 87 people. And ninety percent of those people were really screaming because they wanted direction in life. |
| V11 | FGD (Plan 3000) | Leader(Plan3000)1 | We are not asking for support, so that they do nothing, that they do not do any activity, but that they - within their possibilities as PWD- start to do something for themselves, to strengthen themselves. And we, as leaders, go around controlling, so that they can achieve that. That would be the objective, as I said at the beginning “we have to give them the fishing rod to fish, not the fish to eat” |
| V12 | FGD (Valledupar) | PWD(Valledupar)1 | For me it was very difficult, because until 2019 I had been working for six years with senior citizens and I had my own small entrepreneurship. I was a street vendor, which was my son's livelihood or my family's livelihood, it helped my household. As a result of the pandemic, everything stopped. There were no options for me to sell my product. I lost my job and I lost the opportunity I had to work. |
| V13 | FGD (Plan 3000) | Leader(Plan3000)2 | This man, who got his little corner shop to polish shoes, is now no longer with us. He is now with God. The pandemic has taken him. |
| V14 | FGD (Neiva) | Caregiver(Neiva)1 | The wheelchair has been God's little hand to the Lord. I can also be with her. I have my little business and well, thanks to the Lord, it is now easier, because if I go to work, I have no one to leave her with. So, for me it has been a great help in my business. |
| V15 | FGD (Neiva) | PWD(Neiva)2 | Also my brother (who is also as a PWD part of the project) was a beneficiary witht the production of gelatine and now he is work in his project, right? It's a little bit, but even if it is a little bit, he now collaborates with us. |
| V16 | FGD (Várzea Grande) | PWD(Várzea Grande)1 | Our main focus (within a newly founded organization after the project) was on reforming the wheelchair sector and trying to adapt and bring change to hospitals. (…) We also provide service to other wheelchair users and assist people with disabilities in the health unit. My co-worker (…) goes from neighborhood to neighborhood, checks the health units, checks and then provides service. |
| V17 | FGD (Valledupar | PWD(Valledupar)1 | And to learn more: we have learned that people with disabilities have rights, we have duties; that previously people with disabilities were not taken into account and this association has opened more inclusion for people with disabilities. |
| V18 | FGD (Valledupar) | PWD(Valledupar)1 | We now have more rights or we know more rights. (…) Because the truth is, that I, I as a person with disability, previously would go to an EPS (health insurance entity) or an institution and they would tell me, "Sit down, stay there and wait." (…) Nowadays I won't. (…) I speak with right in the EPS or any institution that I arrive. |
| V19 | FGD (Neiva) | Leader(Neiva)1 | The board made up of 10 (PWD) who chose us, thank them! Because of them, I represent all the PWD, because I want to achieve; to fulfill each one of their dreams or to make a project for each one of the people to be happy, joyful and to live well. That's what I want with all my heart. I give my heart to them, to everyone, because I want them to be motivated. I am the motivator for the people, for the disabled. |
| V20 | FGD (Várzea Grande) | PWD(VárzeaGrande)1 | If you give a voice to everyone, a person with disability can give voice to its neigbourhood and try to improve it. That's what was done and very well done. They were able to arrange a meeting at the right time in the neighborhood. There have been some small changes. |
| V21 | FGD (Várzea Grande) | PWD(VárzeaGrande)1 | This motivated me to try to start to study in college, to try to take that idea of empowerment and apply it in everyday life. So much so that I started my own business and even more tried to lobby for small attitude changes in my neighborhood. Small changes in your community's can make a difference |
| V22 | FGD (Várzea Grande) | Leader(Várzea Grande)1 | They are people who think about the other, it's not just about them. And that is what we bring, and because we have a community that wants to participate, we have large groups. |
| V23 | FGD (Neiva | Leader (Neiva)1 | I want to thank everyone for this project. Every person I already in the project here with their life - I see a vision for them - they deserve it. |
| V24 | FGD (Neiva) | Caregiver(Neiva)1 | We are proud that we have us. As I said before: we are privileged. |
| V25 | FGD (Neiva) | Caregiver(Neiva)1 | With the store and with the magazines I have done well. Thanks to the Lord, I have shoes, I have clothes. (…) For one of my sisters-in-law, who are coffee makers, I send lotions, cream, shampoo, and it is very good for me. |
| V26 | FGD (Várzea Grande) | Leader (Várzea Grande)1 | We couldn't find support in this project, we were unable to move forward on anything. |
| V27 | FGD (Valledupar) | Caregiver(Valledupar)1 | The organizer taught us to value ourselves, to love ourselves as the mothers that we are, first of all, as the women that we are and to be valued (…).The self-esteem that we had, let's say, was in a certain way very low. She made us believe that as people with disabilities we have values, that we have the opportunity to get ahead: She taught us to grow and to value ourselves and to grow and to see a better future.. |
| V28 | FGD (Várzea Grande) | PWD(VárzeaGrande)1 | XX (name omitted due to privacy) who did an excellent job to from that group, to create a management structure where everybody was directed by action. (…) XX is also wonderful. So much that I called XX to partner with us there, to be part of the board (Of the newly founded NGO for PWDs). |
| V29 | FGD (Neiva | Caregiver(Neiva)1 | Well, the truth, the truth is that I joined with doubts, because I had mistrust, because it meant a lot of sacrifice. But then I found myself starting to talk to my friends and everything and things became clear. |
| V30 | FGD (Várzea Grande) | Leader(VárzeaGrande)1 | When you are part of GLRA and XXX (name omitted due to privacy) said something, everybody had enormous respect. Because GLRA is an NGO that it respects in our state. It is respected. |
| V31 | FGD (Neiva ) | Caregiver(Neiva)1 | I have had difficulties because this pandemic has really hit us hard, but with the help of God and of you, because you have been the goal for us, or rather the little light that has taken us there. Thanks to our leader, who, thanks to the Lord, we already have as a leader, and she has been a warrior at our side. So first of all we thank God and you and then we are going to go forward with God and the Virgin and see if we can get through the difficulties. |
| V32 | FGD (Plan 3000) | Leader(Plan3000)2 | Well, the truth is that everyone has his/her opinion on what did not work out well, but this project has to continue! This one! We're going to continue. |
| V33 | FGD (Valledupar) | PWD(VárzeaGrande)1) | But we will continue among ourselves with the people who are closest to us online. Let's change this project. Let's see how we are going to help these people who are in need now, because that is the question in the middle of the pandemic. |
| V34 | FGD (Várzea Grande) | Leader(VárzeaGrande)2 | Everybody did their best possible, we did, but as (Leader1) says: it didn´t start on a good basis. GLRA needs to re-analyze and needs to focus more on the people, because I think that’s it. |
| V35 | FGD (Várzea Grande) | Leader(VárzeaGrande)2 | I also see it this way - it's not the coordinators` fault, but it is the fault of this group that formed in three countries. If it (the methodology) worked well in a country, it doesn´t mean it is good for all countries. |
| V36 | FGD (Várzea Grande) | Leader(VárzeaGrande)2 | I didn´t understand the project (…). I could not understand the project, because for me it was a different methodology from what I knew from previous projects. |
| V37 | FGD (Várzea Grande) | Leader(VárzeaGrande)2 | Mato Grosso is champion in leprosy. We need to  fight leprosy, we have to give workshops. We have to visit people. We have to go to other communities. We have to visit other neighboring municipalities. So we have to act in the State, showing, speaking. This is not only the task of the government. (…)What is needed, is to work more on leprosy, as we had been doing before. |
| V38 | FGD (Várzea Grande) | Leader(VárzeaGrande)1 | We also get scared because it even could have an impact on GLRA and it begins to be discredited, and that is a great fear for us. (…) it's a work of 20 - 30 years or more. |
| V39 | FGD (Várzea Grande) | PWD (VárzeaGrande)1 | This hasbeen really good and it's changed my life. In particular, it has changed my life way it has changed other people's lives as well. |
| V40 | FGD (Vàrzeae Grande) | PWD (VárzeaGrande)1 | So only because of lack of funding, some things were left out, that could have done better. They could have done a better job in providing care, but the general idea was good, the basis was good and important for many people. But I think there are a few things in which we should have worked better. |
| V41 | FGD (Vàrzeae Grande) | Leader(VárzeaGrande)1 | Our work has always been to work on leprosy, the one with more focus on leprosy. And that is why the project for you is innovative but there was no time for us to do this assimilation. It was very difficult for the people. |
| V42 | FGD (Várzea Grande) | Leader(VárzeaGrande)1 | I do believe that setting foot in Colombia probably the project worked out, there, due to the issue of the coordinator’s presence. Here we didn't have her presence. And because the Brazilian coordinator lives in an other city. |
| V43 | FGD (Várzea Grande) | PWD(VárzeaGrande)1 | The general idea of the work was good. The base was very good, we tried to leave the close relationsships within the group and tried to open up more to the people. Catch a whole group of people with disabilities. The idea is great and the essence was good, but poorly elaborated. There was a lack of technical training, knowledge was lacking, the logistics were not so good, there were some errors but the idea was good. It was a good idea. |
| V44 | FGD (Vàrzeae Grande) | Leader(VárzeaGrande)1 | If XX arrives to talk to the public, that experience he has, that he studied, is different from what the project coordinator says. Because they're saying, “the coordinator doesn't have any disabilities. She doesn't know what's going on with us day to day.” PWD very much seeks this representativeness |
| V45 | FGD (Plan 3000) | Leader(Plan3000)2 | We agreed that there was a plan to help everyone, right? But XX (name and function omitted due to privacy) got sick, could no longer attend, we could no longer hold the meetings and we could not help other people either. So, the whole project was left there. (…) Because of the pandemic. And because XX (second person) also got sick. |
| V46 | FGD (Plan 3000) | Leader(Plan3000)2 | The truth, look, we haven't had a single meeting last year. We couldn`t, because of the pandemic. Well, just like in the whole country, everything was interrupted. And it is worse for the people. And well, these people who are, well, dedicated. |
| V47 | FGD (Várzea Grande) | PWD(VárzeaGrande)1) | I didn't have direct contact, no (referring to contact with PWD from other countries). |
| V48 | FGD (Valledupar) | Caregiver(Valledupar)1 | When they were calling for participation, when she knew about this project, she also called the people that she knew and that had disabilities. (…) She had the knowledge of her community. |
| V49 | FGD (Neiva) | Leader(Neiva)1 | Some were left out because they didn't - as I tell you - meet the requirements. The disability that you have - like me, the physical and everything - the one from birth. You need to invest resources as well. For the whole process. |
| V50 | FGD (Valledupar ) | Caregiver(Valledupar)1 | There are a lot of people who really can't participate for "x,y" reason, because they chose or because they weren`t enrolled or because they don't know about the project. There are a lot of people who really need that collaboration, but either they didn't know about the project, or they weren't informed or they were not really taken into account. Or because the spaces are limited, it's just extremely limited. |
| V51 | FGD (Valledupar ) | Caregiver(Valledupar)1 | Our leader belongs to the displacement community and well, in a way she had more knowledge of what the people in need. She made a list of the people she knew and passed it on to the association, to the project coordinator, so that she could see the needs of her community. |
| V52 | FGD (Plan 3000) | Caregiver(Plan 3000)1 | The truth is that, we would like - as leaders - to teach the people that they really need: to teach them how to fish, not to give them fish. And in that, the Foundation was helping us a lot, because it gave them a little capital with material so that they can continue working and developing. The truth is that this was the right thing for them, right? |
| V53 | FGD (Várzea Grande) | Leader(VárzeaGrande)1 | But the community can help itself, right. Also with investment from GLRA, but more from the community itself. I visited a house on Thursday. They needed a bathroom, things like that. (…)Then it was - the priest sent the bricklayer but he couldn`t get here because they wanted to do it by themselves. (…)the priest paid the bricklayer and then the person didn't accept it. So, the wife's husband wouldn't accept it. So from that I knew, the lack of acceptance is due to their pride. It is a person who needs and needed it and did not accept help. This also discourages us, because we want to help them, but the person did not accept it. This case made me very sad. |
| V54 | FGD(Neiva) | Leader(Neiva)1 | We decide everything on the board. There are 7 of us. (…) And the community chooses us, we were chosen by vote. |
| V55 | FGD (Várzea Grande) | Leader(VàrzeaGrande)1 | I had never had a case of leprosy in my family. And now, since last year, my granddaughter has been affected by leprosy. She is now being treated for leprosy. What I was talking about is theory, the practice starts now. Obviously, I'm not going through what the other leader (with leprosy) went through. (...) I Know a 6% of what a disabled person goes through. I don't know. I'm not disabled, I don't have any difficulty with that. Unlike PWD1 or Leader2, as I always say. They are admirable people, they are people who go to the fight. |
